# Dual Negativity of CD56 and CD117 Links to Unfavorable Cytogenetic Abnormalities and Predicts Poor Prognosis in Multiple Myeloma

**DOI:** 10.3390/jcm11216524

**Published:** 2022-11-03

**Authors:** Dong Zheng, Mingxia Zhu, Qihui Li, Wenli Wan, Yingtong Chen, Hongmei Jing

**Affiliations:** Department of Hematology, Lymphoma Center, Peking University Third Hospital, 49 Huayuan North Road, Beijing 100191, China

**Keywords:** multiple myeloma, cytogenetic abnormality, prognosis, CD56, CD117

## Abstract

The prognostic value of CD56 and CD117 expression on myeloma cells is controversial. This study aims to analyze the correlation of CD56 and CD117 expression with cytogenetic abnormalities and survival. A total of 128 patients with newly diagnosed multiple myeloma (NDMM) were recruited in this single-center retrospective study. Flow cytometry and FISH tests of marrow cells were performed for all of the subjects. The statistical methods included a chi-squared test, univariate and multivariate COX regressions, and a Kaplan-Meier survival curve analysis. Regarding the cytogenetics, the incidence of IgH/FGFR3 translocation was more frequent in patients with a negative CD56 (*p* = 0.003). CD56 negativity was an independent adverse factor associated with a poor prognosis (*p* = 0.019) and indicated a shorter overall survival (OS) (*p* = 0.021). Patients with dual negative CD56 and CD117 trended toward a poorer OS (CD56^−^CD117^−^ vs. CD56^+^CD117^−^, *p* = 0.011; CD56^−^CD117^−^ vs. CD56^+^CD117^+^, *p* = 0.013). In conclusion, CD56 is a prognostic marker that independently affects OS and is associated with adverse cytogenetic abnormalities. Patients with a dual negativity of CD56 and CD117 have a worse clinical outcome.

## 1. Introduction

Multiple myeloma (MM) is the second most common hematologic malignancy and is characterized by anemia, hypercalcemia, renal insufficiency, osteolytic destruction, and possible damage to target organs [1]. Over the past ten years, MM treatments have advanced significantly, and the ten-year relative survival for patients has improved from 18.1% to 34.9% [2]. However, MM is still an incurable illness and a complex biological disease [3]. As a result, both risk stratification and prognostic assessments in MM are necessary. The vital prognostic factors include blood biochemical and cytogenetic markers but do not contain antigenic indicators of malignant plasma cells.

CD56 acts as an approximately 140KD neural cell adhesion molecule (NCAM) and is a membrane glycoprotein commonly expressed by neural tissues, NK cells, and T cells [4]. CD56 is connected to tumor cells and it is most prominently expressed in MM cells but not in healthy plasma cells [5]. Previous studies found that the malignancy probability of MM was linked to low CD56 expression [6,7]. Moreover, the hematopoietic growth factor receptor CD117, which exhibits tyrosine kinase activity, is primarily expressed in myeloid progenitor cells and is not present in normal cells [8,9]. CD56 and CD117 are only present in the myeloma population, which makes them “tumor-associated markers” compared with normal plasma cells.

Considering that the functions of CD56 and CD117 with cytogenetic abnormalities and survival in MM have not been thoroughly analyzed, in this work, we retrospectively investigated the plasma cell results of 128 NDMM patients under treatment with flow cytometry (FCM) and chromosomal fluorescence in situ hybridization (FISH). With the help of statistical methods, the correlation of CD56 and CD117 with cytogenetic abnormalities and survival was further discussed. This study can provide clinical value for evaluating the cytogenetic and prognostic importance of CD56 and CD117 in MM.

## 2. Materials and Methods

### 2.1. Patients

A total of 128 NDMM patients were hospitalized at the Department of Hematology at Peking University Third Hospital from January 2017 to June 2022. The diagnosis of MM was verified by the criteria set out by the International Myeloma Working Group (IMWG). In each case, 4 mL of bone marrow was aspirated through the posterior superior iliac crest, anticoagulated with heparin, and then exposed to FISH and FCM tests within 24 h. After the medical research ethics council at Peking University Third Hospital accepted the study protocol, each participant signed an informed consent form. (Ethics code: M2021665).

The patients’ baseline clinical information includes the following: hemoglobin, serum calcium, blood creatinine, β2-microglobulin (β2-MG), lactate dehydrogenase (LDH), albumin, blood immunoglobulin-fixed electrophoresis, light chain protein, and the proportion of malignant plasma cell infiltration in the bone marrow. For all of the patients, MM was prognostically risk-stratified using the ISS, R-ISS, DS, and mSMART stratification. The patients received 4–8 cycles of treatment, including bortezomib- and/or lenalidomide-based therapies and traditional induction chemotherapy schemes. After four courses of treatment, the doctors assessed the therapeutic effects, dividing them into stringent complete response (sCR), complete response (CR), very good partial response (VGPR), partial response (PR), stable disease (SD), and progressive disease (PD). Telephone consultations and outpatient records followed all of the participants through 30 June 2022.

### 2.2. FCM

Five tubes of bone marrow samples from each patient were tested using the following four-color fluorescent antibody combinations: FITC/PE/Per CP5.5/APC, CD38/CD200/CD45/CD138, CD38/CD117/CD45/CD56, CD20/CD19/CD45/CD38, cytoplasmic Kappa/cytoplasmic Lambda/CD45/CD138, and isotype control. Each tube contained 100 µL of the specimens.

A total of 50,000 cells were collected and evaluated to obtain the combined CD45/side-angle scattered light intensity (SSC) and CD38/SSC gates, which allowed for the identification of the plasma cells. The positive rate of various antigens was calculated, and an antigen positivity of ≥20% was considered positive.

### 2.3. FISH

The following FISH findings are related to high-risk chromosomal changes: t (4;14), t (14;16), t (14;20), del 17p, and gain 1q (p53 mutation). The IgH (14q32) translocations involve chromosomal loci (4p16, 16q23, 20q11, and 11q13), which are connected to IgH partners (FGFR3, MAF, MAFB, and CCND1). Refer to [10] for information on del 17p (TP53), gain 1q (CKS1B), amplification 1q (CKS1B), and del 13q (RB1, D13S319). At least 100 plasma cells from each probe set were analyzed, and at least three abnormal cells had to be present to consider a positivity (gain means three copies; amplification means more than three copies).

### 2.4. Statistical Analysis

The statistical analysis used SPSS 26.0. The difference between the two groups was analyzed using a chi-square test, a *t*-test, and a Mann–Whitney U test. The PFS was defined as the time between the diagnosis of the disease and its progression, recurrence, or death. The OS was described as the time from the diagnosis of the illness to the final follow-up or death. The Cox proportional risk retrospective model was used for univariate and multivariate analyses, and the Kaplan-Meier survival curve analysis was used for the survival analysis. Statistically significant differences were indicated by *p* < 0.05.

## 3. Results

### 3.1. Clinical Characteristics

The 128 NDMM patients had a median age of 63.5 (56–71) years; 79 (61.72%) were men and 49 (38.28%) were women, with a male-to-female ratio of 1.61, as shown in Supplementary Table S1. Table 1 displays the baseline clinical features of the 128 MM patients. The dual negativity of CD56 and CD117 was significantly associated with bone marrow plasma cell infiltration (CD56^−^: *p* = 0.000; CD117^−^: *p* = 0.017). The CD56 negative patients were mainly in ISS stage III (64.1%). However, the age, sex, hemoglobin, blood calcium, albumin, renal insufficiency (creatinine value of >177 µmol/L), LDH, β2-MG, immunoglobulin subtypes, illness stage, and prognostic classification did not differ significantly. Six patients could not be analyzed because they received less than four cycles of the treatment. Only 1 patient was induced with a traditional chemotherapy regimen, and the other 121 patients were induced with bortezomib- and/or lenalidomide-based regimens.

### 3.2. Cytogenetic Abnormalities

All of the patients underwent a FISH analysis. A total of 51.6% (66/128) of the patients had high-risk cytogenetic anomalies, according to mSMART3.0 [11]. The CD56^−^ patients had a significantly higher high-risk cytogenetic incidence (64.1% vs. 46.1%, *p* = 0.060), but there was no significant difference in the CD117^−^ patients (53.8% vs. 48.0%, *p* = 0.518). The IgH/FGFR3 translocation had a high frequency (20.5% vs. 3.4%, *p* = 0.003) and the IgH/CCND1 had a low prevalence (7.7% vs. 23.6%, *p* = 0.034). (Table 2). In addition, we divided CD56 and CD117 into the following four groups: CD56^−^CD117^−^, CD56^+^CD117^−^, CD56^+^CD117^+^, and CD56^−^CD117^+^, corresponding to 29, 49, 40, and 10 patients, respectively. The D13S319 loss, RB1 loss, IgH/FGFR3, and IgH/MAF showed significant differences (*p* = 0.021, *p* = 0.030, *p* = 0.003, and *p* = 0.006, respectively). The IgH/FGFR3 occurred frequently and accounted for 45.5% in the CD56^−^CD117^−^ group. The RB1 and D13S319 losses were common and represented 51.3% and 51.4%, respectively, in the CD56^+^CD117^−^ group (Table 3).

### 3.3. The Impact of Antigen Expression on Patient Survival

**Figure 1:**
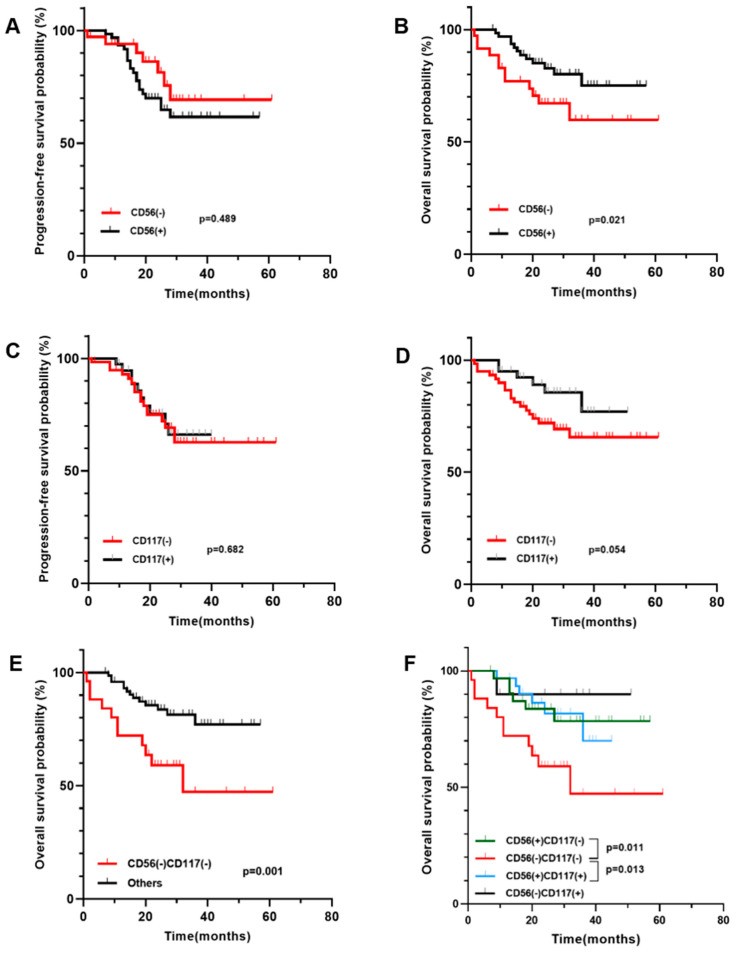
Kaplan-Meier curves for OS and PFS based on CD56 and CD117 expression. (**A**,**B**) CD56^−^ had a significantly shorter OS. (**C**,**D**) CD117 did not affect PFS or OS. (**E**) The CD56^−^CD117^−^ group exhibited a considerably shorter OS. (**F**) CD56^−^CD117^−^ showed a significantly shorter OS.

A total of 24 patients were lost to follow-up in total. The median follow-up period was 24 months (the range was 0–61 months). CD56^−^ indicated a worse OS (*p* = 0.021, Figure 1B). In addition, we separated CD56 and CD117 into the following four groups: CD56^−^CD117^−^, CD56^+^CD117^−^, CD56^+^CD117^+^, and CD56^−^CD117^+^, corresponding to 29, 33, 32, and 10 patients, respectively. The CD56^−^CD117^−^ group had a considerably shorter OS. (CD56^−^CD117^−^ vs. CD56^+^CD117^−^, *p* = 0.011; CD56^−^CD117^−^ vs. CD56^+^CD117^+^, *p* = 0.013; Figure 1F).

### 3.4. Univariate Analyses and Multivariate COX Regression Analyses for Prognostic Factors

According to the univariate analysis, an ISS stage III, a β2-MG value of >5.5 mg/L, a CD56^−^, and an LDH value of >250 U/L were significantly associated with adverse PFS and OS, respectively (Table 4). In terms of the multivariate analysis, a CD56^−^ and an elevated LDH were independent indicators significantly affecting OS (Table 5).

## 4. Discussion

MM is a malignancy with an abnormal proliferation of monoclonal plasma cells that is still incurable and has a fatal relapse outcome. Most studies have used flow cytometry and immunohistochemistry to analyze CD56 and CD117 because these methods are relatively inexpensive and widely available, and the positive antigen expression threshold is normally 20% or 50% [12,13,14]. The results may be presented differently if the positive threshold is altered. This study utilized a 20% antigen-positive threshold with flow cytometry. Multiparametric flow cytometry (MFC) allows for the simultaneous evaluation of surface and intracytoplasmic antigens in contrast to immunohistochemical analyses. Additionally, it offers a shorter return time and enables a measurable residual disease (MRD) assessment [5,15,16]. As a result, we believe that flow cytometry is a useful technique for evaluating MM tumor-associated markers.

It is commonly accepted that flow cytometry can successfully distinguish benign and malignant plasma cells based on antigen expression. Normal plasma cells are consistently positive for CD19, CD45, CD38, and CD138 but are negative for CD56 and CD117. In contrast, MM cells often express CD38 and CD138 and inconsistently or never express CD56 and CD117 [14,17,18]. Prior research revealed that almost two-thirds of MM patients exhibited CD56 positivity [19], and roughly one-third of MM patients showed CD117 positivity [20]. In the work, 69.5% of MM patients exhibited CD56 positivity and 39.1% showed CD117 positivity, which is broadly consistent with previous studies.

Prior studies have focused on the relationship between the MM risk factors and the expression of CD56 and CD117. In particular, unfavorable cytogenetic abnormalities, higher LDH, higher serum β2-MG, higher anemia, higher incidence of BMPC, and higher frequency of renal failure are all thought to indicate a sign of disease progression [14,21,22]. In our study, there were significant amounts of BMPC infiltration in the groups of CD56 negativity and CD117 negativity.

The prognostic impacts depend on the critical cytogenetic abnormalities in MM. The intricacy of the bone marrow microenvironment, oncogene overexpression, and genomic instability further complicate the cytogenetic abnormalities [23]. However, the cytogenetic information is insufficient in previous research; it prevented accurately capturing the link with tumor antigen expression [12,21]. The IgH/FGFR3 translocation is ectopically formed by t (4; 14) (p16; q32) in MM, which frequently indicates a poor prognosis [24]. The IgH/FGFR3 translocation was shown to occur more frequently in CD56^−^ patients than in CD56^+^ patients (20.5% vs. 3.4%, *p* = 0.003). Notably, most of these patients were CD117^−^ (8 out of 11 cases). This demonstrates that a specific molecular biogenetic aberration may be reflected by CD56^−^CD117^−^, a new subgroup of IgH/FGFR3 gene fusions. Therefore, we used survival analysis to compare the CD56^−^CD117^−^ with the other three groups, and we discovered that the CD56^−^CD117^−^ group had a significantly worse OS. According to prior research, the dual negativity of CD56 and CD117 indicates a poor prognosis [7,18,25]. In this study, CD56^−^ was identified as an independent prognostic factor for a poor outcome. Patients with dual negative CD56 and CD117 tended toward a worse OS. According to all of these findings, the poor prognosis for MM is associated with the dual negativity of CD56 and CD117.

Losses of RB1 and D13S319 occur in the deletion of 13q14. Although the deletion of 13q14 alone has less influence on the prognosis [26], the biallelic inactivation of RB1 occurs more frequently, contributing to high risk [27]. Uncertainty surrounds the function of D13S319 biallelic changes. Studies have discovered that the loss rates of RB1 and D13S319 in NDMM are comparable (about 40%) because they are located in the same place on the same chromosome [28]. In this study, although the RB1 and D13S319 loss rates were similar among the four groups, CD56^+^CD117^−^ accounted for the highest percentage, roughly 50%. This group did not have the worst short-term survival; therefore, they should be closely monitored and concentrate on their long-term prognosis.

The analysis has the following limitations: first, it was a single-center retrospective study with a few participants; second, the dynamic changes in antigen expression should have been evaluated during the treatment, but not enough data were available.

## 5. Conclusions

In conclusion, CD56 is a prognostic marker that affects OS independently and is linked to adverse cytogenetic abnormalities. Patients with dual negativity of CD56 and CD117 have an inferior clinical outcome. Additionally, more prospective research is required to further comprehend the roles of tumor-associated markers in MM pathogenesis.

## Figures and Tables

**Table 1:** MM patients with baseline characteristics linked to CD56 and CD117 expression.

Characteristics	CD56^−^	CD56^+^	*p*	CD117^−^	CD117^+^	*p*
	*n* = 39	*n* = 89		*n* = 78	*n* = 50	
Age ≥ 60years (*n*)	28	59	0.539	50	37	0.242
Gender, male (*n*)	20	59	0.108	43	36	0.055
Type of myeloma (*n*)			0.054			0.603
IgG	14	48		37	25	
IgA	9	24		21	12	
IgM	0	1		1	0	
IgD	1	0		0	1	
Light chain	14	14		16	12	
Nonsecretory	1	2		3	0	
DS stage III (*n*)	33	74	0.979	61	46	0.101
ISS stage III (*n*)	25	45	0.336	43	27	0.940
R-ISS stage III (*n*)	9	20	0.510	21	8	0.302
mSMART High-risk (*n*)	17	40	0.887	37	20	0.409
renal insufficiency (*n*)	10	23	0.981	16	17	0.089
Hb (Mean ± SD), g/L	98 ± 23	105 ± 27	0.166	105 ± 25	100 ± 28	0.375
Calcium (median), mmol/L	2.33	2.31	0.903	2.31	2.32	0.988
Albumin (median), g/L	36.4	35.5	0.660	34.9	37.5	0.068
β2-MG (median), mg/L	6.52	5.12	0.184	5.52	5.90	0.794
LDH (median), U/L	201	173	0.073	181	177	0.579
BMPC (median), %	48.00	16.00	0.000	24.50	16.00	0.017
Bone lesions (*n*)			0.701			0.771
Group A	8	21		17	12	
Group B	31	68		61	38	
Response ≥ PR (*n*)	31	72	0.853	63	40	0.915

Cr, creatinine; Hb, hemoglobin. BMPC, bone marrow plasma cell; Renal insufficiency, creatinine value of >177 µmol/L; Bone lesions, according to the imaging examination results (CT, MRI, PET-CT); Group A, osteolytic destruction at one anatomical site and/or diffuse osteoporosis; Group B, osteolytic destruction at more than one anatomical site and/or pathological fractures.

**Table 2:** Cytogenetic abnormality showed by CD56 and CD117 expression.

	CD56^−^	CD56^+^	*p*	CD117^−^	CD117^+^	*p*
	*n* = 39	*n* = 89		*n* = 78	*n* = 50	
D13S319 loss	11(28.2)	24(27.0)	0.885	24(30.8)	11(22.0)	0.277
RB1 loss	11(28.2)	28(31.5)	0.713	26(33.3)	13(26.0)	0.379
IgH/CCND1	3(7.7)	21(23.6)	0.034	12(15.4)	12(24.0)	0.223
CKS1B gain	7(17.9)	23(25.8)	0.404	20(25.6)	10(20.0)	0.475
CKS1B amplification	4(10.3)	7(7.9)	0.445	6(7.7)	5(10.0)	0.491
High risk	25(64.1)	41(46.2)	0.060	42(53.8)	24(46.0)	0.518
1q21 gain and amplification	11(28.2)	31(34.8)	0.462	27(34.6)	15(30.0)	0.587
Del 17p	4(10.3)	6(6.7)	0.492	7(9.0)	3(6.0)	0.739
IgH/FGFR3	8(20.5)	3(3.4)	0.003	8(10.3)	3(6.0)	0.526
IgH/MAF	2(5.1)	0(0.0)	0.091	0(0.0)	2(4.0)	0.151
IgH/MAFB	0(0.0)	0(0.0)	-	0(0.0)	0(0.0)	-
p53 mutation	0(0.0)	1(1.1)	1.000	0(0.0)	1(1.1)	0.391

High risk, according to mSMART 3.0. The high-risk genetic abnormalities include the following: t (4;14), t (14;16), t (14;20), del 17p, p53 mutation, and gain 1q; Del 17p: p53 loss.

**Table 3:** Cytogenetic abnormality categorized by CD56 and CD117 expression.

	CD56^−^ CD117^−^	CD56^+^ CD117^−^	CD56^+^ CD117^+^	CD56^−^ CD117^+^	*p*
	*n* = 29	*n* = 49	*n* = 40	*n* = 10	
D13S319 loss	6(17.1)	18(51.4)	6(17.1)	5(14.3)	0.021
RB1 loss	6(15.4)	20(51.3)	8(20.5)	5(12.8)	0.030
IgH/CCND1	2(8.3)	9(37.5)	12(50.0)	1(4.2)	0.097
CKS1B gain	5(16.7)	13(43.3)	10(33.3)	2(6.7)	0.606
CKS1B amplification	4(36.4)	2(18.2)	5(45.5)	0(0.0)	0.606
1q21 gain and amplification	9(21.4)	16(38.1)	15(35.7)	2(4.8)	0.790
Del 17p	2(20.0)	5(50.0)	1(10.0)	2(20.0)	0.229
IgH/FGFR3	5(45.5)	3(27.3)	0(0.0)	3(27.3)	0.003
IgH/MAF	0(0.0)	0(0.0)	0(0.0)	2(100.0)	0.006
IgH/MAFB	0(0.0)	0(0.0)	0(0.0)	0(0.0)	-
p53 mutation	0(0.0)	0(0.0)	1(100.0)	0(0.0)	0.559

**Table 4:** Univariate and multivariate COX regression analyses for PFS.

Variables	Univariate Analysis	*p*	Multivariate Analysis	*p*
	HR (95%CI)		HR (95%CI)	
CD56^−^ vs. CD56^+^	1.332 (0.586–3.026)	0.494		
CD117^−^ vs. CD117^+^	1.174 (0.542–2.545)	0.684		
Age ≥ 60 vs. > 60 years	1.395 (0.614–3.169)	0.427		
LDH ≥ 250 vs. > 250U/L	1.000 (0.380–2.632)	1.000		
β2-MG ≥ 5.5 vs. > 5.5 mg/L	2.565 (1.090–6.037)	0.031	4.566 (0.000–1.456)	0.929
ISS stage III vs. I + II	2.433 (1.034–5.726)	0.042	0.001 (0.000–1.742)	0.937
Cytogenetics high risk vs. other	1.850 (0.879–3.895)	0.105		

**Table 5:** Univariate and multivariate COX regression analyses for OS.

Variables	Univariate Analysis	*p*	Multivariate Analysis	*p*
	HR (95%CI)		HR (95%CI)	
CD56^−^ vs. CD56^+^	2.409 (1.113–5.213)	0.026	2.529 (1.165–5.489)	0.019
CD117^−^ vs. CD117^+^	2.381 (0.956–5.932)	0.062		
Age ≥ 60 vs. > 60 years	1.516 (0.036–3.612)	0.348		
LDH ≥ 250 vs. < 250U/L	2.540 (1.130–5.706)	0.024	2.694 (1.195–6.073)	0.017
β2-MG ≥ 5.5 vs. < 5.5 mg/L	1.116 (0.512–2.429)	0.783		
ISS stage III vs. I + II	1.234 (0.560–2.719)	0.602		
Cytogenetics high risk vs. other	2.030 (0.931–4.426)	0.075		

## Data Availability

The data of this research is not available in public but it can be obtained from the corresponding authors upon reasonable request.

## Supplementary Materials

The following supporting information can be downloaded at: https://www.mdpi.com/article/10.3390/jcm11216524/s1, Figure S1: Kaplan-Meier curves for overall survival (OS) and progression-free survival (PFS) based on CD200 and light chain expression. Table S1: Clinical characteristics of patients with newly diagnosed multiple myeloma. Table S2: Characteristics of patients with CD56 positive and IgH/FGFR3 translation positive. Table S3: Cytogenetic abnormality and genes affected.
