# Supplementary material for: Dual Negativity of CD56 and CD117 Links to Unfavorable Cytogenetic Abnormalities and Predicts Poor Prognosis in Multiple Myeloma
Source: J Clin Med. 2022 Nov 3;11(21):6524. doi: 10.3390/jcm11216524 (PMC9653854; doi:10.3390/jcm11216524)
Supplement: Supplementary file 1 [file jcm-11-06524-s001.zip › jcm-1951839-supplementary.pdf]

Table S1. Clinical characteristics of patients with newly diagnosed multiple myeloma

Characteristics	Number (%) or median (range)
Age	63.5 (56-71)
Gender, male/female	79/49
Hemoglobin, g/L	103±26
LDH, U/L	179 (146-229)
Creatinine, umol/L	93 (78-179)
Calcium, mmol/L	2.31 (2.22-2.55)
Albumin, g/L	35.85 (30.03-40.80)
β2-MG, mg/L	5.58 (3.20-10.42)
ISS stage, I: II: III	32:26:70
R-ISS stage, I: II: III	24:75:29
DS stage, I: II: III	7:14:107
mSMART, standard-risk: high-risk	71:57
IgG: IgA: IgM: IgD: NS: light-chain	62: 33: 1: 1: 3: 28
BMPC, %	20 (1.7-93.5)

ISS: International staging system; DS: Durie-Salmon; mSMART: Mayo Stratification of MyelomaAnd Risk-adapted Therapy; NS: nonsecretory; BMPC: bone marrow plasma cell

Table S2. Characteristics of patients with CD56 positive and IgH/FGFR3 translation positive

Characteristics	CD56(+) and IgH/FGFR3(+) n=3
CD117(-)	3(100)
D13S319 deletion	3(100)
RB1 deletion	3(100)
CKS1B amplification	2(66.7)
IgH/CCND1	1(33.3)
1q21 amplification	2(66.7)
P53 deletion	1(33.3)
IgH/MAF	0(0.0)

Table S3. Cytogenetic abnormality and genes affected

Cytogenetic abnormality	Genes affectd
Del 13q	RB1 and D13S319
Gain 1q	CKS1B
Del 17p	TP53
t(4;14)	FGFR3
t(11;14)	CCND1
t(14;14)	c-MAF
t(14;20)	MAFB

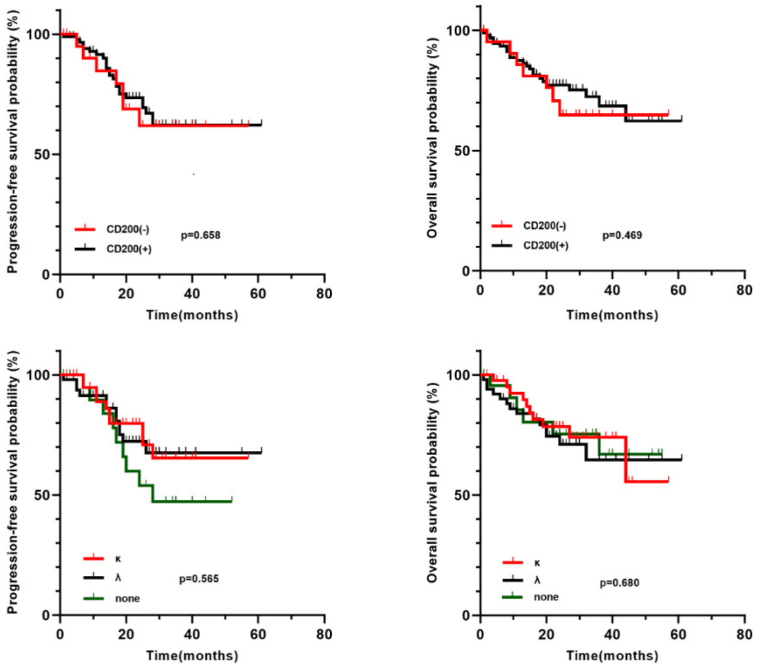

Figure S1. Kaplan–Meier curves for overall survival (OS) and progression-free survival (PFS) based on CD200 and light chain expression
